# Accelerated somatic mutation calling for whole-genome and whole-exome sequencing data from heterogenous tumor samples

**DOI:** 10.1101/gr.278456.123

**Published:** 2024-04

**Authors:** Shuangxi Ji, Tong Zhu, Ankit Sethia, Wenyi Wang

**Affiliations:** 1 Department of Bioinformatics and Computational Biology, The University of Texas MD Anderson Cancer Center, Houston, Texas 77030, USA;; 2 NVIDIA Corporation, Santa Clara, California 95051, USA

## Abstract

Accurate detection of somatic mutations in DNA sequencing data is a fundamental prerequisite for cancer research. Previous analytical challenges were overcome by consensus mutation calling from four to five popular callers. This, however, increases the already nontrivial computing time from individual callers. Here, we launch MuSE 2, powered by multistep parallelization and efficient memory allocation, to resolve the computing time bottleneck. MuSE 2 speeds up 50 times more than MuSE 1 and eight to 80 times more than other popular callers. Our benchmark study suggests combining MuSE 2 and the recently accelerated Strelka2 achieves high efficiency and accuracy in analyzing large cancer genomic data sets.

Cancer arises and evolves by accumulating various types of genetic alterations, such as single-nucleotide variation (SNV), copy number alteration (CNA), and structural variation (SV). The high-throughput sequencing (HTS) technology has revolutionized the way we look at many human diseases, particularly cancer. With its constantly improved capacity and reduced cost, HTS is enabling investigations of genetic alterations within large human patient cohorts, hence advancing both basic and translational cancer research. Many computational tools have been developed for calling somatic variants (Xu 2018), which typically require, as input, whole-genome sequencing (WGS) or whole-exome sequencing (WES) data from the tumor tissue, as well as from the blood of the patient to serve as the germline control. WGS provides the most comprehensive coverage to sequence both protein-coding and noncoding regions across the entire genome, whereas WES provides an efficient alternative to WGS by targeting only protein-coding regions that account for 1%–2% of the genome (Alfares et al. 2018), hence achieving both higher read depth (Barbitoff et al. 2020; Sun et al. 2021) and lower sequencing cost.

We previously launched MuSE 1 (Fan et al. 2016), a statistical approach for somatic mutation calling, in which we introduced a combination of nucleotide base-specific Markov substitution model for molecular evolution and a tumor sample–specific error model to estimate tier-based cutoffs for selecting SNVs. Because of its high sensitivity and specificity, especially for calling subclonal SNVs, MuSE 1 was adopted in multiple pipelines, including as a major contributing caller to reach final consensus calls by The Cancer Genome Atlas (TCGA) PanCanAtlas project (Ellrott et al. 2018), across approximately 13,000 tumor samples, and the International Cancer Genome Consortium Pan-Cancer Analysis of Whole Genomes (ICGC-PCAWG) initiative (The ICGC/TCGA Pan-Cancer Analysis of Whole Genomes Consortium 2020), across approximately 2700 tumor samples.

One major limitation of MuSE 1, like many other mutation callers (Koboldt et al. 2012; Larson et al. 2012; Cibulskis et al. 2013), is the computational speed. It takes 2–3 d to finish running the WGS data of a tumor–normal pair on a typical Linux server with an Intel Xeon processor and >100 gigabytes (GB) random access memory (RAM), which explains the commonly seen long wait times for completing mutation calling before any downstream analysis in large patient cohort studies. Here, we present MuSE 2, which maintains the same input, output, and mathematical model as MuSE 1 but accelerates significantly for both WES and WGS data by adopting a new algorithmic programming backbone. MuSE 2 uses a multithreaded producer–consumer model and the OpenMP library for parallel computing, including parsing and uncompressing reads from binary sequence alignment/map formatted (BAM) files, detecting and filtering variants, and writing output. It is also optimized by adopting a more efficient memory allocator. In this paper, we have benchmarked the accuracy of MuSE 2 against three somatic mutation callers, namely, MuTect2 (Cibulskis et al. 2013), SomaticSniper (Larson et al. 2012), and VarScan2 (Koboldt et al. 2012), which are the other highlighted somatic mutation callers in the National Cancer Institute Genomic Data Commons (GDC) DNA-seq analysis pipeline (https://docs.gdc.cancer.gov/Data/Bioinformatics_Pipelines/DNA_Seq_Variant_Calling_Pipeline/), as well as a recently accelerated mutation caller Strelka2 (Kim et al. 2018). We use the consensus mutation calls generated by previous consortial studies with three to five unaccelerated callers (Craig et al. 2016; Ellrott et al. 2018; The ICGC/TCGA Pan-Cancer Analysis of Whole Genomes Consortium 2020). Here, we show the improved utility of our new caller using WES data generated from five tumor–normal pairs and WGS data generated from seven tumor–normal pairs.

## Results

### Overview of approach

**Figure 1.**
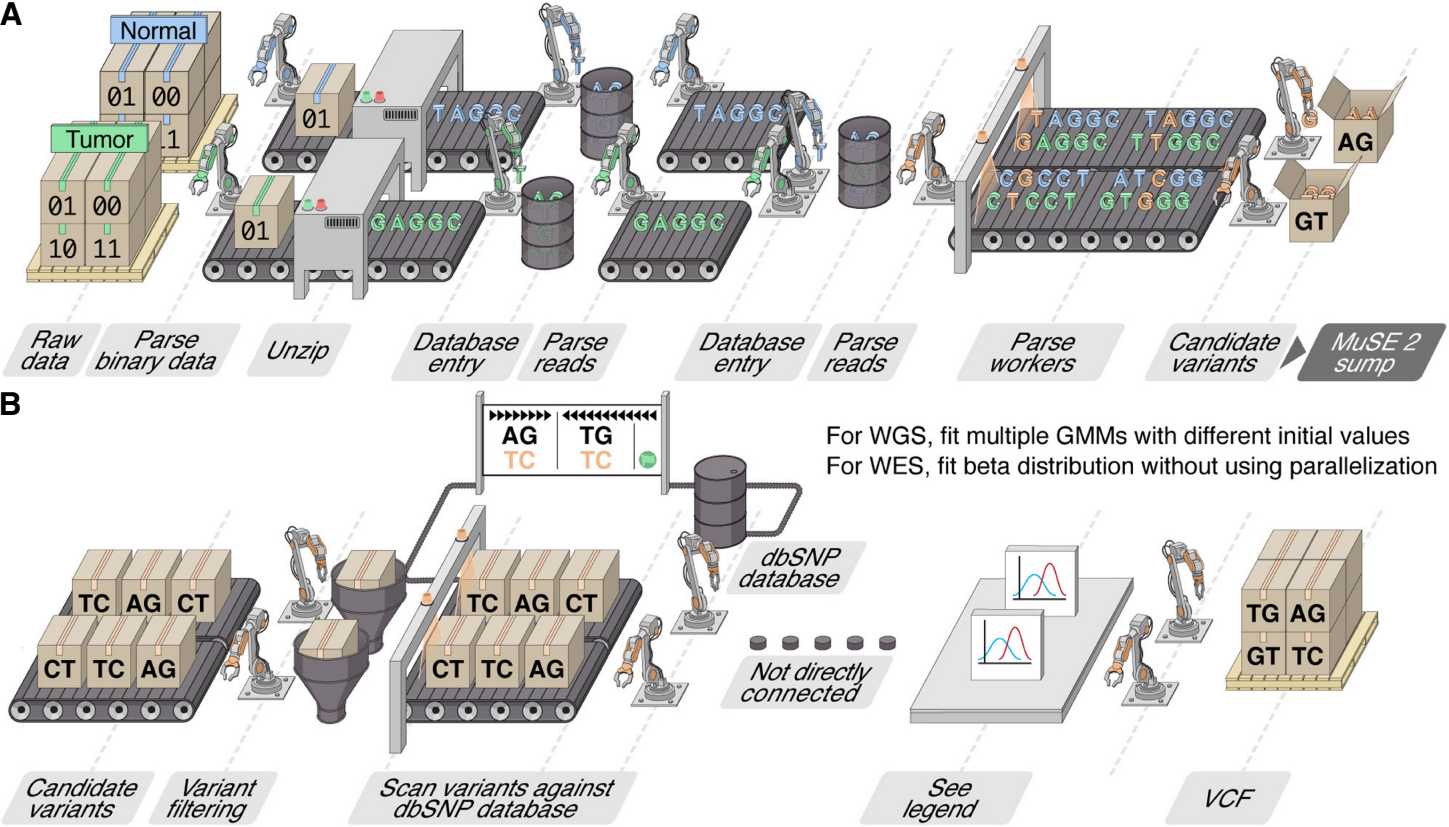
Assembly line illustration of the multistep parallelization implemented in MuSE 2. (*A*) “MuSE call”: Workers (threads) keep fetching chunks from the input BAM files from the tumor and normal samples and unzipping them to the text format of reads. Downstream workers combine the reads from the tumor and normal samples and send to a queue; from there, other workers detect candidate variants. (*B*) “MuSE sump”: Multiple workers are used to take the candidate variants and their corresponding estimated summary statistic π’s and scan them against the dbSNP database, labeling those appearing in the database. For candidate variants from the WGS data, we fit two-component Gaussian mixture models (GMMs) with multiple initializations, distributed to multiple workers, in order to separate true variants from background noise; for candidate variants from the WES data, no parallelization is implemented owing to computational simplicity as we simply fit a Beta distribution to π’s.

MuSE 2 takes as input the indexed reference genome FASTA file, the BAM format sequencing data from a pair of tumor–normal tissues (Supplemental Fig. S1), and the dbSNP (Sherry et al. 2001) variant call format (VCF) file, which is bgzip-compressed, tabix (Li 2011)-indexed using the same reference genome. Unlike MuSE 1, which can only use one core, MuSE 2 takes advantage of the multicore resources in a modern computer or a computing node for somatic SNV calling from WES/WGS data (Fig. 1A,B).

Because our benchmarking study requires a large number of computational resources to cover multiple callers and scenarios, we only include results for the WES data from five tumor–normal pairs and for the WGS data from five tumor–normal pairs, which are randomly selected and downloaded from the GDC data portal and the ICGC data portal, respectively. The sequencing depths from these samples reflect the wide ranges presented in both data sets (Supplemental Table S1; Supplemental Fig. S2). We further include WGS data from two tumor–normal pairs to evaluate mutation calling performances on newer sequencing platforms.

We compare the SNV entries in the output VCF files generated by MuSE 2 with those by MuSE 1 for each patient sample with the same or a different number of CPU cores. Because each SNV entry is denoted by one line of string in a VCF file, we compare the strings from both methods line by line. The result shows that all the entries from the two methods are identical with the same number or different number of cores (Supplemental Fig. S3).

### Accuracy benchmarking for real tumor samples

**Figure 2.**
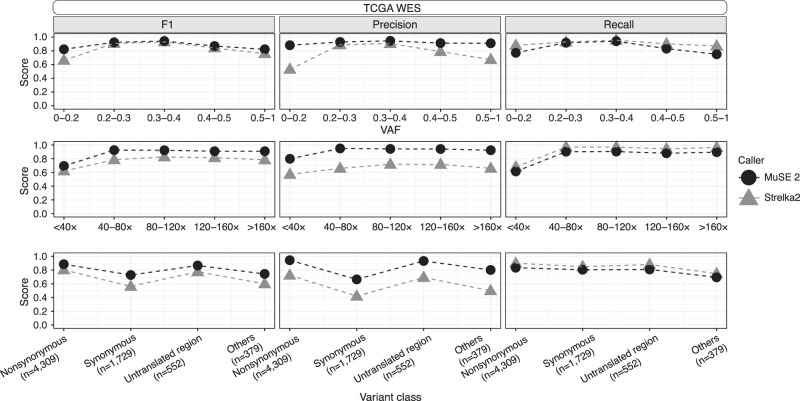
Comparisons of F1 score, precision, and recall between MuSE 2 and Strelka2 within each bin of variant allele frequency (VAF; *top*), sequencing read depth (*middle*), or variant class (*bottom*) for TCGA WES data. The calls of each method and the consensus calls, which are used as the truth set, are pooled from the WES data of five patient samples in TCGA.

**Figure 3.**
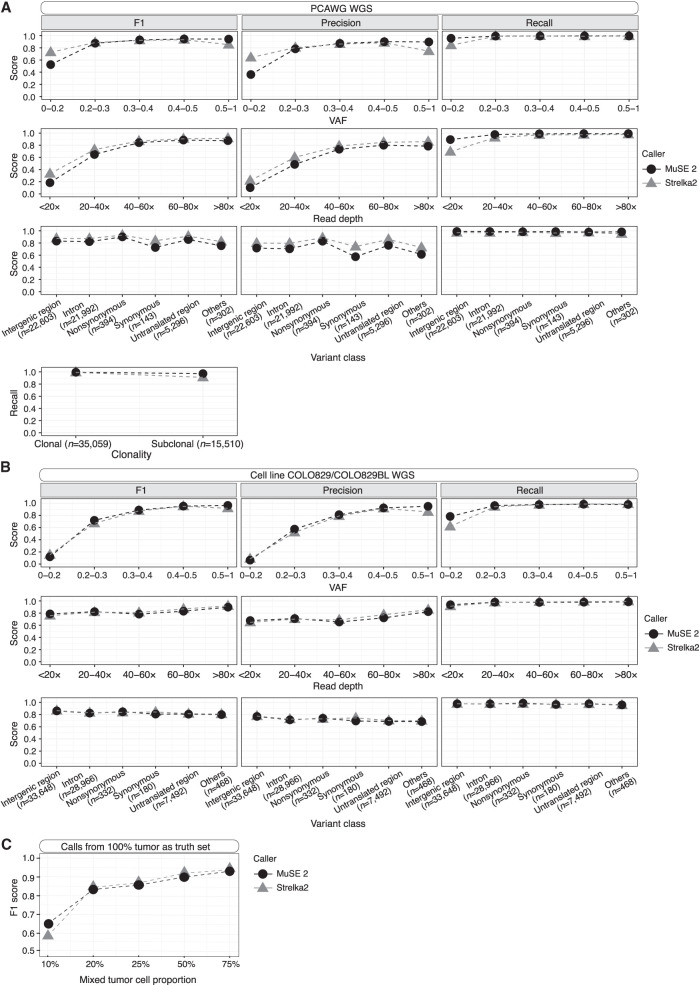
Accuracy benchmarking of MuSE 2 and Strelka2 within each bin of VAF (*first* row), sequencing read depth (*second* row), or class of variant effects (*third* row) for PCAWG WGS (*A*) and cell line WGS (*B*) data. Comparison of recall between the two methods within different clonality for PCAWG WGS data is shown in the last row of *A*. The calls of each method, as well as the truth set, are pooled from the WGS data of the selected five patient samples from PCAWG (*A*) or the WGS data of the cell line COLO829/COLO829BL generated from two platforms (*B*). The number of consensus calls for a variant class is included in the *x*-axis labels. (*C*) F1 scores of MuSE 2 and Strelka2 with varied tumor cell proportions. Calls from the tumor purity of 100% were used as the truth set.

We evaluate the performance of MuSE 2 and compare it to other callers using the consensus SNV calls from TCGA (for the WES data) and PCAWG (for the WGS data) as truth sets. The truth sets include 168–2553 somatic SNVs (mean = 1394, median = 932) for the WES data and 3813–19,081 somatic SNVs (mean = 10,146, median = 8073) for the WGS data. We first compared among callers whose predecessors contributed to the consensus call: MuSE 2, MuTect2, SomaticSniper, and VarScan2 for TCGA WES and MuSE 2 and MuTect2 for PCAWG WGS. We divided the mutation positions into multiple bins defined by variant allele frequency (VAF), or sequencing read depth, and by classes of variant effects or clonality (Methods) and calculated the precision, recall, and F1 score, that is, the harmonic mean of precision and recall for each bin. For both the WES and WGS data, MuSE 2 achieves a higher precision at a similar or higher recall, hence a higher F1 score (Supplemental Fig. S4; for TCGA, see A; for PCAWG, see B) across all bins of VAF and read depth and across variant classes. It also achieves a higher recall for subclonal consensus SNV calls from PCAWG WGS (Methods) (Dentro et al. 2021). We then compared the performance between MuSE 2 and Strelka2 (Kim et al. 2018). Strelka2 used machine learning and curated data to train a position-specific error score, and was developed after the release of PCAWG consensus calls. In contrast to machine learning, MuSE 2 uses the same Bayesian Markov model as MuSE 1 to explicitly define an evolutionary process and estimate model-based parameters based on the data at hand. Overall, Strelka2 performs well in both WES and WGS data. Compared with MuSE 2, its performance is lower in WES (Fig. 2) across all bins of VAF, read depth, and different variant class. However, in the case of WGS data, it either matches or surpasses MuSE 2 in precision at a similar recall rate (Fig. 3A). There is an exception that Strelka2 still shows a slightly lower recall than MuSE 2 at positions with low VAF (<0.2) or low read depth (<20×).

### Accuracy benchmarking for tumor cell line data

As the TCGA and PCAWG data used above were generated by Illumina HiSeq 2000 more than a decade ago, we further obtained a set of tumor cell line sequencing data that was generated using newer sequencing platforms to compare the caller performances. The cell line tumor–normal pair COLO829/COLO829BL was used to generate WGS data on HiSeq X Ten, called COLO829 Illumina, and on NovaSeq, called COLO829 10X, and the consensus calls from three mutation callers were generated through a multi-institutional effort (Methods) (Craig et al. 2016). Although the sequencing data were newer, the consensus call effort was relatively old, and the three callers did not entirely match what was established by the TCGA and PCAWG projects. They include MuTect2's predecessor MuTect (Cibulskis et al. 2013), Strelka2's predecessor Strelka (Saunders et al. 2012), and Seurat (Christoforides et al. 2013). We therefore implemented two strategies to benchmark the accuracy of MuSE 2 and Strelka2. First, we took the consensus calls as the truth set, which includes 35,543 SNVs from the two pairs of samples, COLO829 Illumina and COLO829 10X. Second, we put aside the consensus calls and instead used the mutation calls made in the tumor cell line (100%) as the truth set. This truth set then includes 45,853 SNVs from MuSE 2 and 44,257 SNVs from Strelka2, respectively, called from COLO829 Illumina. We then evaluated how many of these initial calls were recovered in a in silico diluted data set in which the tumor cell proportion decreases to 75%, 50%, 25%, 20%, and 10% (Methods). MuSE 2 and Strelka2 did equally well with both truth sets. MuSE 2 presented a slightly higher recall than did Strelka2 at positions with a low VAF (<0.2) or in samples with a low tumor cell proportion (≤10%) (Fig. 3B,C). We note that this slight advantage in MuSE 2 is consistently observed across all WGS data, whereas an advantage of higher precision in MuSE 2 is consistently observed in WES data.

### Speed benchmarking

**Figure 4.**
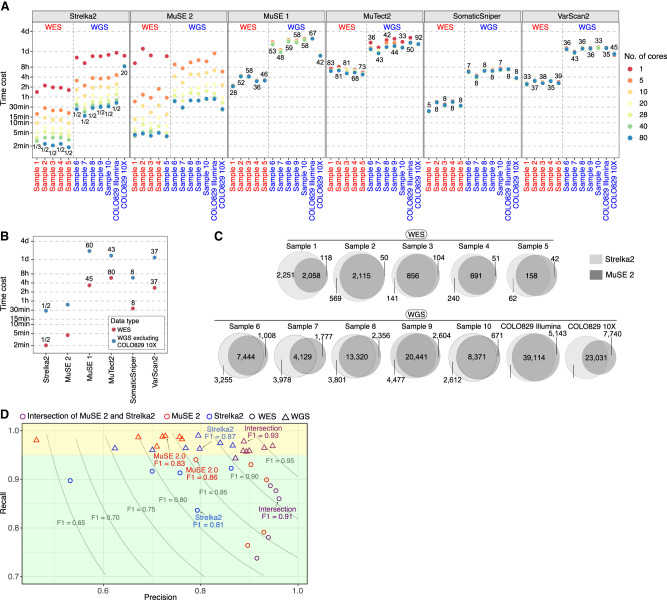
Benchmarking the speed and usability of MuSE 2. (*A*) The runtime of MuSE 2 against MuSE 1 and the other four methods for both the WES and WGS data across different numbers of cores. The numbers in the plot represent the fold speedup of MuSE 2 (with 80 cores) relative to the other methods whose time cost is averaged across different numbers of cores (excluding core = 1). For Strelka2, only the time cost with 80 cores is considered. (*B*) A simplified version of *A* in which the time cost of each method is averaged across all samples (excluding COLO829 10X as an outlier; see *A*) and different numbers of cores. (*C*) Venn diagrams showing the unique and shared SNV calls of MuSE 2 and Strelka2. (*D*) Scatter plot of the precision and recall for the intersection calls from MuSE 2 (in red) and Strelka2 (in blue) and the calls from each of the two methods (in purple) against the previously reported consensus calls, which are considered as the benchmark. For both the WES (circle) and WGS (triangle) data, the median F1 scores of the intersection calls and calls of each individual method are shown. Two shaded rectangles highlight the difference of the performance metrics between the WES and WGS data. Results from the WGS data are located in the *top* rectangle.

We compare the speed of running MuSE 2 against MuSE 1, MuTect2, SomaticSniper, VarScan2, and Strelka2 on a computing cluster. Each method is tested with the number of CPU cores at one, five, 10, 20, 28, 40, and 80. All methods are assigned with the same RAM of 50 GB for the WES data and 150 GB for the WGS data. The time cost of each method for each pair of data is shown in Figure 4A. Except for COLO829 10X, both MuSE 2 and Strelka2 continue to gain computational advantages with an increasing number of CPU cores, whereas the other four methods do not. We examine the overall speed performances of these methods with MuSE 2 at 80 cores and Strelka2 at 80 cores, as well as the average time cost across multiple runs over the different numbers of cores except for core = 1 (for which the computing resource is too limited) for the other methods (Fig. 4B). Both MuSE 2 and Strelka2 achieve much faster SNV calling compared with all the other methods. For the WES data, MuSE 2 accelerates 28–58 times (mean = 44) compared with MuSE 1, 68–83 times (mean = 77) compared with MuTect2, five to eight times (mean = 8) compared with SomaticSniper, and 33–39 times (mean = 36) compared with VarScan2. Similarly, for the WGS data, it accelerates 48–59 times (mean = 57) compared with MuSE 1, 33–44 times (mean = 41) compared with MuTect2, seven to eight times (mean = 8) compared with SomaticSniper, and 33–43 times (mean = 37) compared with VarScan2. On the other hand, Strelka2 is faster than MuSE 2 for all the WES data and the WGS data except for COLO829 10X. It is about a twofold speedup on average compared with MuSE 2. For COLO829 10X, however, Strelka2 stopped gaining speed after five CPU cores, whereas MuSE 2 continued accelerating; its computing time is 20 times that of MuSE 2 at 80 cores.

Overall, MuSE 2 and Strelka2 are the two top accelerated methods compared with the others in the above benchmarking. We further examine the difference between the SNV calls reported by them for the same patient sample (Fig. 4C). For the WES data, 46%–78% (mean = 66%) of the calls are identified by both; 2%–16% (mean = 7%) of the calls are unique to MuSE 2; and 13%–51% (mean = 27%) of the calls are unique to Strelka2. For the WGS data, 41%–77% (mean = 62%) of the calls are identified by both; 6%–18% (mean = 11%) of the calls are unique to MuSE 2; and 13%–45% (mean = 26%) of the calls are unique to Strelka2.

We further investigate the feasibility of using the intersect calls from these two methods to reproduce the consensus calls for these data generated by previous studies (Craig et al. 2016; Ellrott et al. 2018; The ICGC/TCGA Pan-Cancer Analysis of Whole Genomes Consortium 2020). For the WES data, these calls notably improve the F1 scores of Strelka2 calls, while maintaining a comparable F1 score with the MuSE 2 calls (Fig. 4D; Supplemental Table S2). This suggests that running MuSE 2 alone might be sufficient for the WES data when the computing resource is limited. For the WGS data, on the contrary, Strelka2 calls (0.76–0.92) have higher F1 scores than do MuSE 2 calls (0.63–0.88) in all the patient samples, whereas the intersection calls outperform the two individual callers and reach the highest F1 scores (0.91–0.96). Also, the intersect calls achieve the highest precision values (0.92–0.96 for WES, 0.87–0.95 for WGS) for all the data benchmarked, despite the differences of read depths and sequencing platform they were generated (Supplemental Table S1). The intersect calls maintain good recall values at 0.74–0.89 (median = 0.86) for the WES data and at 0.96–0.98 (median = 0.96) for the WGS data. The recall values of either the intersect call sets or the individual call sets from the two methods are consistently higher for the WGS data than the WES data (Fig. 4D, all results from the WGS data fall in the top rectangle). Also, MuSE 2 call sets achieve higher precisions and F1 scores and lower recalls for WES but achieve higher recalls and lower precisions and F1 scores for the WGS data compared with the calls from Strelka2. Because the F1 scores of intersect calls are lower in WES than in WGS, we further investigated whether the inclusion of a third caller could improve the accuracy for WES. This led to an interesting observation that adding VarScan2 or SomaticSniper as a third caller did not improve precision, recall, or F1, whereas adding MuTect2 improved recall but decreased precision and F1 (Supplemental Table S3). In the project of multicenter mutation calling in multiple cancers (MC3) organized by TCGA, the latter strategy was further expanded to include five callers, maximizing recall, and was followed by a postfiltering pipeline, independent of any callers, to improve precision (Ellrott et al. 2018). This postfiltering pipeline removes potentially false-positive variants that are caused by germline contamination, sequence artifacts, low read depth in the normal sample, and nonexonic variants. In summary, combining mutation calls from the two accelerated callers MuSE 2 and Strelka2, for example, by simply taking an intersection of the calls, is promising to achieve optimized mutation calling in a significantly shorter wait time. This strategy is particularly useful for the WGS data and for analysis of large patient cohorts. With the WES data, running MuSE 2 alone can be a cost-effective strategy to obtain mutation calls with high precision and a reasonable F1 score.

### A Snakemake pipeline for somatic SNV calling

Finally, we introduce a fully automated mutation calling pipeline for general users who do not have the time or expertise to learn about the nuances in optimizing mutation calling accuracy, using the Snakemake workflow management system (Supplemental Fig. S5) (Köster and Rahmann 2012). This user-friendly pipeline allows for running all preprocessing steps, MuSE 2 and Strelka2 for mutation calling, and all postprocessing including the consensus steps, in the background without manual curation. It is compatible with typical Linux systems and computing clusters and optimizes memory and CPU use by parallelizing independent tasks.

## Discussion

Precision medicine and personalized cancer treatments have advanced remarkably in the past decade, which greatly benefited from the accurate identification of genetic variations in the tumor tissue using HTS data. An efficient and accurate somatic mutation caller is crucial to the scientific studies of all cancers and their clinical management. Previously the accuracy and utility of MuSE 1, either alone (Fan et al. 2016) or as a member of a multicaller consensus calling strategy, have been validated by multiple consortial projects (Ellrott et al. 2018; The ICGC/TCGA Pan-Cancer Analysis of Whole Genomes Consortium 2020). This study further develops MuSE 2 in order to fully use resources on a high-performance computing machine, including both the CPU cores and memory allocation. The producer–consumer model behind the parallelization implemented in the step of “MuSE call” gives MuSE 2 the ability to manage multiple processes (workers) at the same time: They run independently at their own rates without being affected by the computing load of other processes. Because the calculation in the step of “MuSE sump” is more straightforward (the computing speed bottlenecks only reside in several for-loop iterations), we use the OpenMP library, with which the parallelization is relatively trivial. The speed-up by MuSE 2 becomes evident when it is run on at least four to five cores to take advantage of the multistep parallelization. In summary, MuSE 2 improves the mutation calling utility of MuSE 1 by accelerating its computing speed by up to 50–60 times for both the WES and WGS data. MuSE 2 reduces the computational time cost of a somatic mutation calling project from ∼40 h to <1 h for the WGS data and from 2–4 h to ∼5 min for the WES data, from each pair of tumor–normal samples.

MuSE 2 is much faster than the other three benchmarked callers, namely, MuTect2, SomaticSniper, and VarScan2. It is slightly slower than Strelka2 for the sequencing data generated by HiSeq 2000 and HiSeq X Ten but is much faster than the latter with NovaSeq. Because we only include one pair of tumor–normal WGS data from HiSeq X Ten and NovaSeq, respectively, more data are needed to validate this result in the future study. The intersection of MuSE 2 and Strelka2 calls can substantially improve precisions without much loss in recalls, hence improving the overall F1 scores for both the WES and WGS data benchmarked. We therefore show the utility of the intersection calls from these two fast callers compared with using each caller individually or using unaccelerated callers.

In contrast to the current consensus calls of TCGA and PCAWG and the cell line study, running MuSE 2 and Strelka2 to generate intersect calls may greatly improve the efficiency of genomic data analysis for large patient cohorts, especially for those with WGS data. We also found running MuSE 2 alone is a cost-effective solution for mutation calling in WES data, as it would otherwise require four to five callers plus postfilterings to achieve much higher recall and precision. Finally, in order to improve accessibility by general users, we provide a Snakemake workflow pipeline that automatically runs preprocessing, intersect mutation calling using the two accelerated callers, and postprocessing without human intervention. We note that the hg19 genome assembly was used throughout the study because all consensus calls were based on hg19. Given the underlying models of MuSE 2 and Strelka2, we expect the performance of the variant calling of both methods to be insensitive to genome assemblies. As the switch of assembly build from hg19 to hg38 can impact preprocessing and read mapping to generate the input data, some difference in variant calls could be observed, which should not substantially affect the conclusions (Gao et al. 2019). Future development of MuSE 2 includes indel calling and SNV calling from formalin-fixed paraffin-embedded (FFPE) samples and from tumor samples only, all of which require an advanced error model construction, as well as further benchmarking. In summary, we expect the proposed MuSE 2 to significantly accelerate the variant calling process and benefit the cancer research and clinical communities.

## Methods

### Sample selection

The consensus mutation calls of the TCGA portion of the PCAWG project were downloaded from the ICGC data portal (https://dcc.icgc.org/releases/PCAWG/consensus_snv_indel). The consensus mutation calls of the TCGA MC3 project were downloaded from the NCBI Genomic Data Commons (GDC) (https://gdc.cancer.gov/about-data/publications/mc3-2017). We randomly selected five patient samples from each of the two repositories and downloaded the BAM files from https://dcc.icgc.org/repositories and https://portal.gdc.cancer.gov/analysis_page?app=Downloads, respectively. The sequencing data of these 10 patient samples were generated by Illumina HiSeq 2000. We further downloaded BAM files of WGS data from the metastatic melanoma cell line COLO829 and the matched normal lymphoblastoid line COLO829BL from the European Nucleotide Archive (ENA; accession code PRJEB27698; https://www.ebi.ac.uk/ena/browser/view/PRJEB27698). We downloaded the latest WGS data set (Espejo Valle-Inclan et al. 2022). The sequencing libraries either were prepared with Illumina TruSeq Nano reagent kit and sequenced on the HiSeq X Ten platform (COLO829 Illumina) or were prepared on the 10x Chromium platform and sequenced on the NovaSeq platform (COLO829 10X). For COLO829 Illumina, we also downloaded the BAM files of the cell line with mixed tumor cell proportions of 75%, 50%, 25%, 20%, and 10%. These data were simulated by in silico mixing of reads from COLO829 (100% tumor) and COLO829BL (normal sample) with different ratios.

### BAM preprocessing

MuSE 2 adopts the same preprocessing steps for the unaligned sequencing reads of the tumor–normal pair as MuSE 1, which include trimming poor-quality bases, removing adapters, marking duplicate reads, performing local indel realignment for the paired tumor–normal BAM files jointly, and recalibrating base quality scores (Supplemental Fig. S1). In this study, the sequencing reads are aligned against the hg19 reference genome build using BWA-MEM (Li 2013).

### Sequencing depth

The sequencing depth of each BAM file after preprocessing is estimated by SAMtools (Li et al. 2009) with the “depth” command. For the WGS data, the overall depth was calculated as the average of the read depths of all genomic locations. For the WES data, the overall depth was calculated as the average of the read depths of the genomic locations in the exon regions defined by the exome capture kit downloaded from GDC (https://gdc.cancer.gov/about-data/publications/mc3-2017).

### Parallel computing implementation for MuSE

#### MuSE call

We implement a multithreaded producer–consumer model that deploys threads for parsing and uncompressing reads from BAM files, for variant filtering and detection, for writing outputs, and for monitoring the whole process. The model connects all the threads concurrently by thread-safe queues and atomic variables. We also adopt a faster and more efficient memory allocator (i.e., TCMalloc: https://github.com/google/tcmalloc) rather than use the default malloc in C and the new in C++ in this step. The parallelization model starts with creating six threads, three for the BAM of the tumor sample and the other three for the BAM of the normal sample: One of the three threads parses the compressed binary data and sends its reference to two queues, namely, ChunkReadQueue and ChunkUnzipQueue; the other two threads take the data from the ChunkUnzipQueue, decompress it, and label it as “processed.” This change is also effective for the data in ChunkReadQueue, because these two queues in fact store the same data. Another thread (i.e., read) is then created, which takes uncompressed data from ChunkReadQueue, recovers them to read format for both the BAM tumor sample and the BAM of normal sample, and pushes them to the same queue, ReadQueue. A new thread named processReads is created; it parses the reads from ReadQueue and sends them to the queue, processQ. *n* threads of named workers are created to take the reads from processQ and process them following the same prefiltering and evolutionary model as MuSE 1. The last thread is named as “monitor,” which prints the sizes of the queues every second. Here, users can specify *n* according to the number of cores available in the input of MuSE 2 (Supplemental Fig. S6).

#### MuSE sump

We use the OpenMP library to parallelize the three most time-consuming parts in MuSE sump. The first is the loading of candidate variants, as well as the corresponding estimates of equilibrium frequencies for all four alleles (A, C, T, G) for each variant from MuSE call, and filtering out the variants whose ratio between the VAF from the normal sample and the VAF from the tumor sample is above a predefined cutoff (0.05) (Fan et al. 2016). The second is scanning for the remaining variants in the dbSNP, marked as “true” or “false” if they appear in the database or not. For the WGS data, MuSE 1 fits a two-component Gaussian mixture model to the allele frequencies of the postfiltered variants to separate true mutations from background noise. The parameters (e.g., mean, standard deviation, and proportion) of the two components are estimated using the expectation-maximization algorithm, which is repeated 50 times with random initializations. For the three parts, we parallel the for-loop iterations using the “omp parallel for” clause from OpenMP in MuSE 2 to deploy the computation on multiple cores.

### Speed benchmarking settings

For all the benchmarked methods, if the number of cores requested lies in {1, 5, 10, 20, 28}, the processor is the Intel Xeon gold 6132 CPU at 2.60 GHz; if the number of cores requested lies in {40, 80}, the processor is the Intel Xeon platinum 8380 CPU at 2.30 GHz. We run each method by submitting the load sharing facility (LSF) job script using the bsub command, with which we can easily control the RAM and the number of cores specified for each method. The options for the six callers can be found in Supplemental Table S4.

### Precision and recall

For the samples from TCGA and PCAWG, we used the consensus SNV calls published previously (Ellrott et al. 2018; The ICGC/TCGA Pan-Cancer Analysis of Whole Genomes Consortium 2020) as a truth set; for the cell line COLO829/COLO829BL, we used the SNV calls downloaded from Craig et al. (2016) as a truth set. The version information of the benchmarked callers is listed in Supplemental Table S5. Indel calls were removed from the call set before any comparison. For the WGS data, we took calls from all tiers in MuSE 2 (Fan et al. 2016), and only calls from the PASS category from the other callers for each patient sample. We filtered out low-quality SNVs from the consensus calls from PCAWG WGS data that are labeled as “LOWSUPPORT,” “OXOGFAIL,” “bSeq,” “bPcr,” “GERM1000G,” “GERMOVLP,” “NORMALPANEL,” or “REMAPFAIL” (https://dcc.icgc.org/releases/PCAWG/consensus_snv_indel). Consistently, we used consensus calls for the cell line COLO829/COLO829BL, which had already gone through a similar postfiltering process (Craig et al. 2016). For the WES data, we selected calls from all the categories except for “Tier5” from MuSE 2 and selected only calls from the PASS category from the other callers for each patient sample. We also used the consensus calls of TCGA WES that had already gone through postfiltering (Ellrott et al. 2018). The intersection between any two sets from the same patient sample was identified by matching the SNV IDs, which combined the columns of CHROM, POS, REF, and ALT from the two VCF files. For the WES data, we removed the SNVs from the intersection calls outside the regions defined by the exome capture kit of TCGA.

We considered any calls reported by the consensus, but not by the intersection calls, as false negatives; any calls reported by the intersection calls, but not by the consensus, as false positives. We calculated precision, recall, and F1 score to evaluate the accuracy of a call set against a truth set.

$$F_{1}=2\times \frac{\mathrm{Precision}\times \mathrm{Recall}}{\mathrm{Precision}+\mathrm{Recall}}.$$

### Definitions of VAF bins, sequencing depth bins, clonality, and variant effect annotation for SNVs

To resolve the issue that the read depth (including the number of reads supporting the reference allele and the alternate allele) can be different for the same SNVs from MuSE 2, Strelka2, and the consensus calls, we used alleleCount (https://github.com/cancerit/alleleCount) to recalculate the read depth and VAF for all the unique SNVs from MuSE 2, Strelka2, and the consensus calls. We finally generated the bins of VAFs (i.e., 0–0.2, 0.2–0.3, 0.3–0.4, 0.4–0.5, and >0.5) and read depths (<40×, 40–80×, 80–120×, 120–160×, and >160× for the WES data, <20×, 20–40×, 40–60×, 60–80×, and >80× for the WGS data) for the calls of each method for detailed comparisons.

We downloaded the consensus subclonal reconstruction results (Dentro et al. 2021) for the consensus calls of PCAWG WGS data from ICGC data portal (https://dcc.icgc.org/releases/PCAWG/subclonal_reconstruction/). Each SNV in the consensus calls is defined as either clonal or subclonal when such information is available. To compare the performance between MuSE 2 and other callers, we restricted the SNVs from each caller overlapping with the ones from the consensus calls such that they can be annotated as clone or subclone. Therefore, only recall is evaluated.

We used Ensembl VEP (v101) (McLaren et al. 2016) to predict the effect of a SNV. For simplicity, we merged nonsense and missense variants into nonsynonymous variants; variants in the 3′ untranslated region (UTR), 5′ UTR, 3′ flank, and 5′ flank into untranslated region variants; variants in splice region, translation start site, and RNA variants into the others category. We also renamed silent variants to synonymous variants. We have four classes for the SNVs from TCGA WES data (nonsynonymous, synonymous, untranslated region, and others) and six classes for the SNVs from PCAWG or cell line COLO829/COLO829BL WGS data (nonsynonymous, synonymous, intergenic region, intron, untranslated region, and others).

### Software availability

MuSE 2 is implemented in C++ and is available at GitHub (https://github.com/wwylab/MuSE) with the GPL-2.0 license. A Dockerfile is included in the repository for building MuSE 2 into a Docker container running on Linux machines. A Snakemake pipeline for somatic SNV calling, MuSE.Snakemake 1.0, is also available on the GitHub repository. The source code of the repository is also available as Supplemental Code.

## Supplementary Material

Supplement 1

Supplement 2

Supplement 3

Supplement 4

Supplement 5

Supplement 6

Supplement 7

Supplement 8

Supplement 9

Supplement 10

Supplement 11

Supplement 12
