# Supplementary material for: Accelerated somatic mutation calling for whole-genome and whole-exome sequencing data from heterogenous tumor samples
Source: Genome Res. 2024 Apr;34(4):633–41. doi: 10.1101/gr.278456.123 (PMC11146589; doi:10.1101/gr.278456.123)
Supplement: Supplement 1 [file Supplemental_Fig_S1.docx]

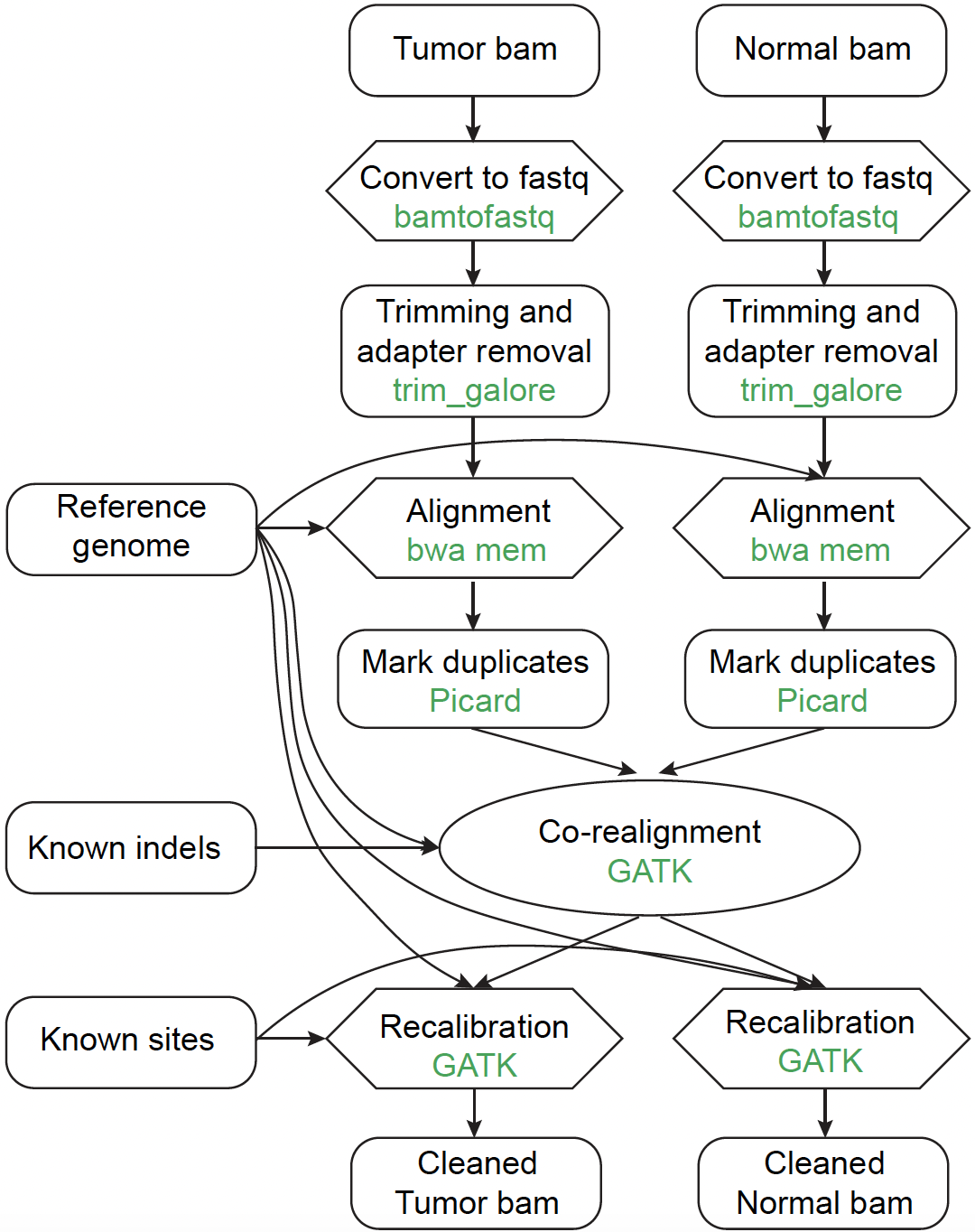


**Supplemental Fig. S1 | Flowchart of sequencing read pre-processing before running a mutation calling method in the benchmarking study.**
