# Supplementary material for: Accelerated somatic mutation calling for whole-genome and whole-exome sequencing data from heterogenous tumor samples
Source: Genome Res. 2024 Apr;34(4):633–41. doi: 10.1101/gr.278456.123 (PMC11146589; doi:10.1101/gr.278456.123)
Supplement: Supplement 2 [file Supplemental_Fig_S2.docx]

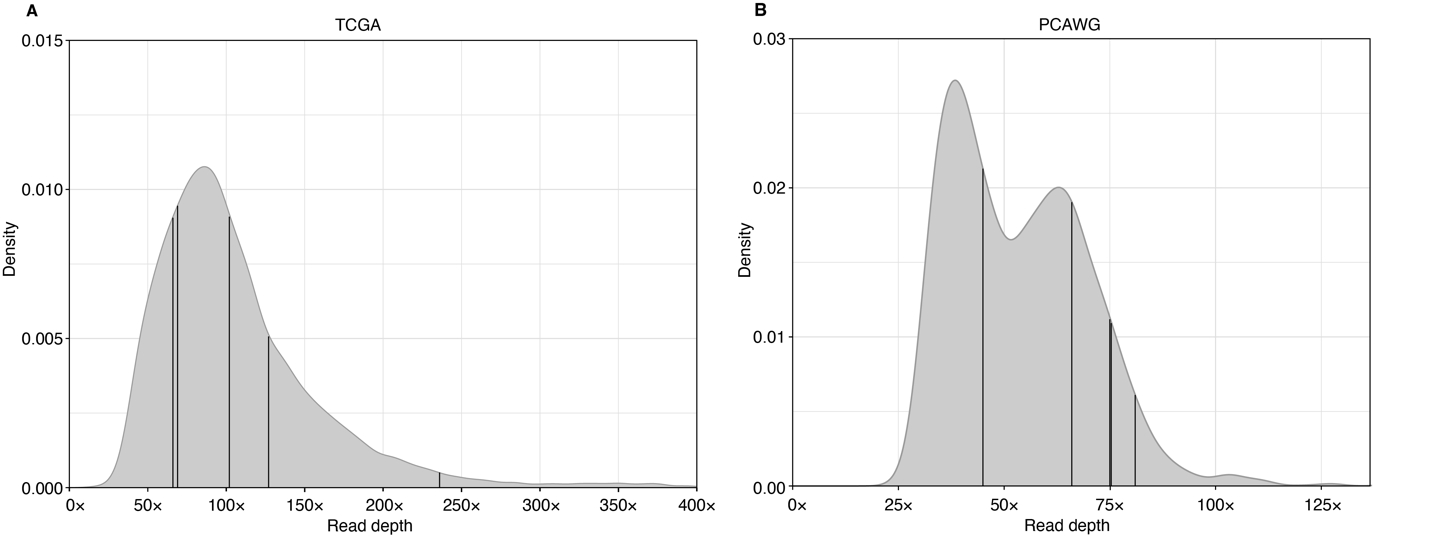


**Supplemental Fig. S2 | Distributions of the average read depths of (A) TCGA WES, (B) PCAWG WGS data of tumor samples are shown by the curves.** Vertical lines represent the selected tumor samples used in this study.
