# Supplementary material for: Accelerated somatic mutation calling for whole-genome and whole-exome sequencing data from heterogenous tumor samples
Source: Genome Res. 2024 Apr;34(4):633–41. doi: 10.1101/gr.278456.123 (PMC11146589; doi:10.1101/gr.278456.123)
Supplement: Supplement 3 [file Supplemental_Fig_S3.docx]

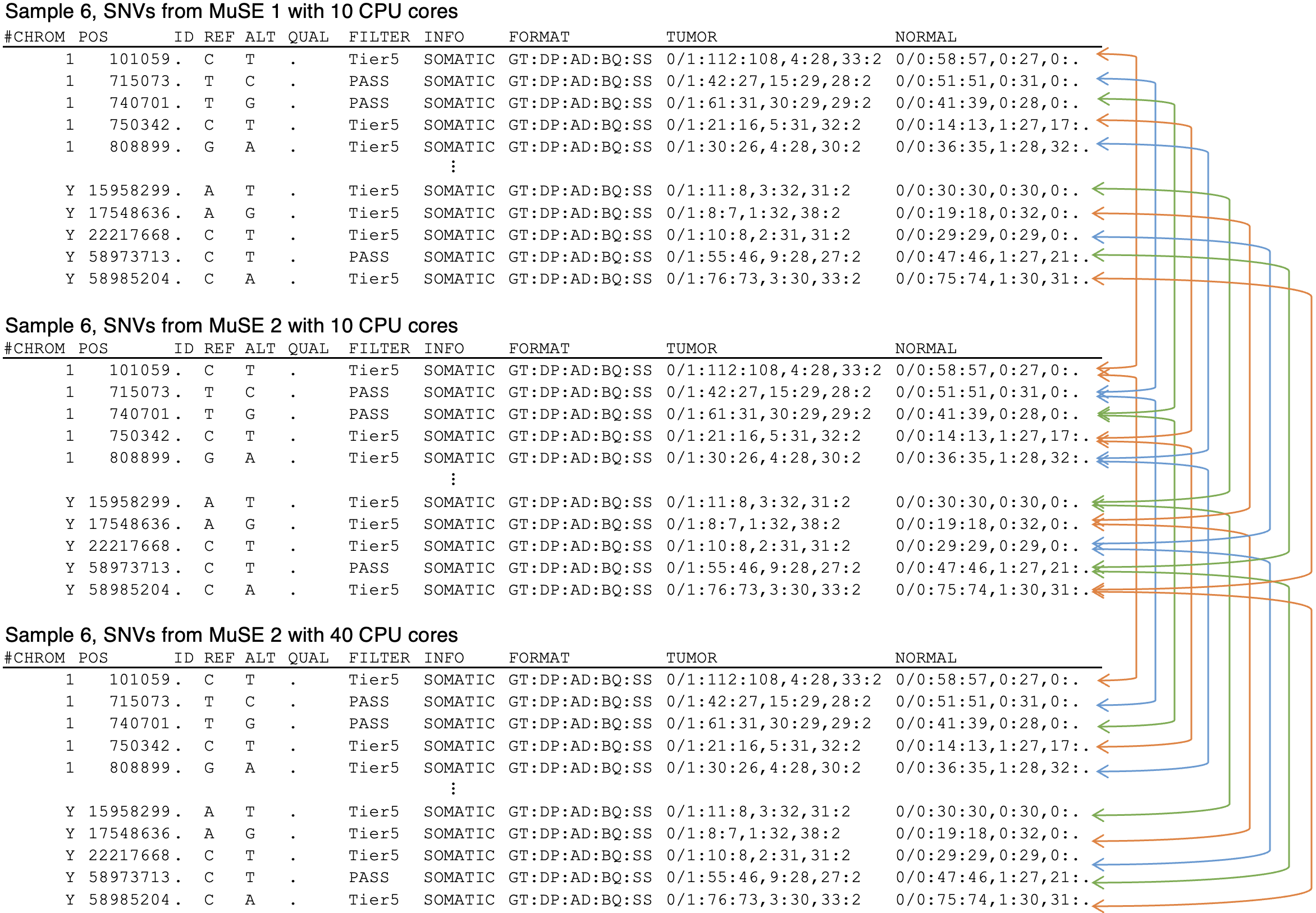


**Supplemental Fig. S3 | Illustration of SNV calling results from MuSE 1 and MuSE 2.** A total of 10 positions in Sample 6 are shown with identical results from running the two versions with different numbers of CPU cores.
