# Supplementary material for: Accelerated somatic mutation calling for whole-genome and whole-exome sequencing data from heterogenous tumor samples
Source: Genome Res. 2024 Apr;34(4):633–41. doi: 10.1101/gr.278456.123 (PMC11146589; doi:10.1101/gr.278456.123)
Supplement: Supplement 4 [file Supplemental_Fig_S4.docx]

**
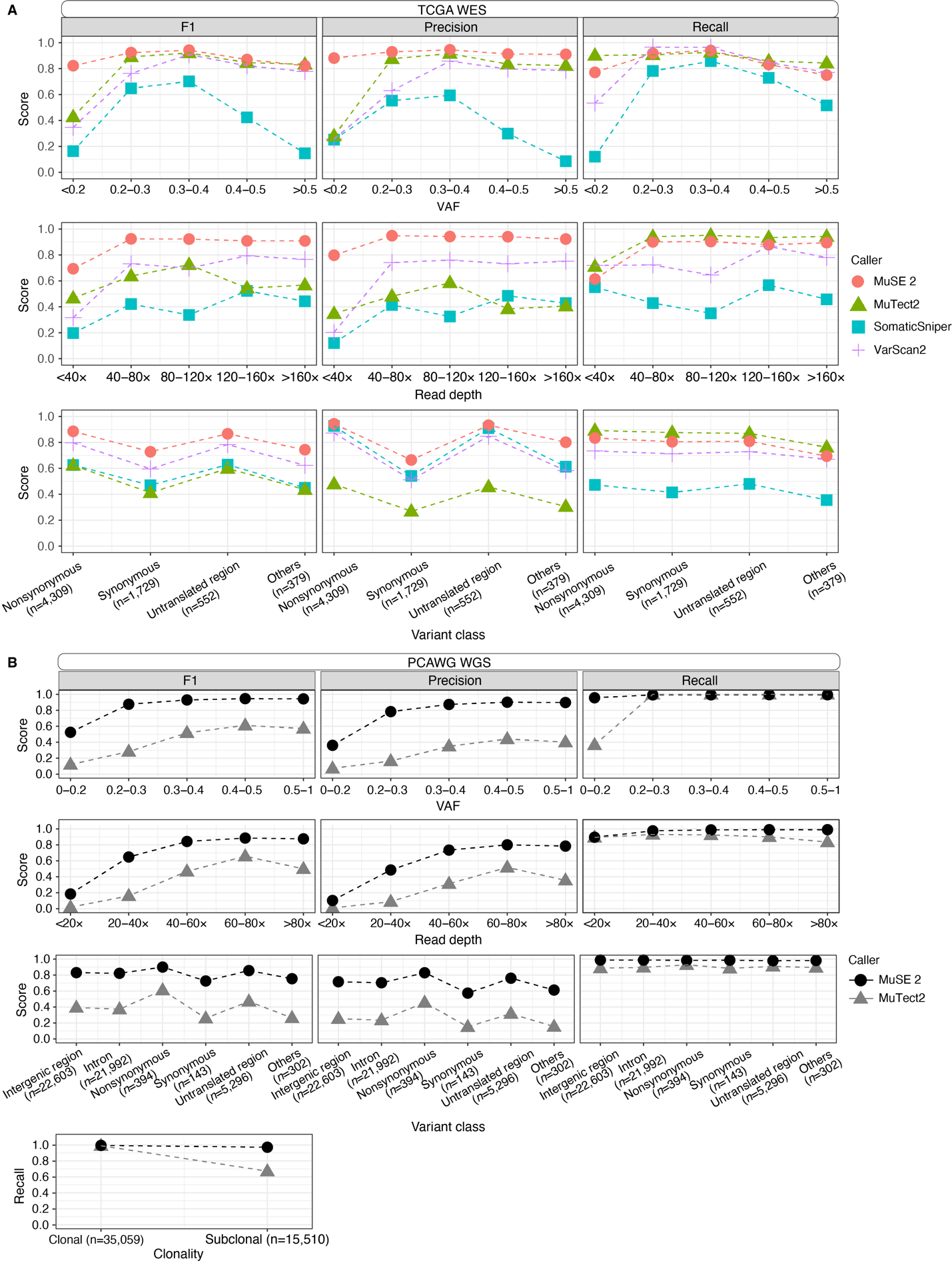
Supplemental Fig. S4 | Accuracy benchmarking of MuSE 2 and other callers used for consensus calls within bins of variant allele frequency (VAF, first row) or sequencing read depth (second row), variant classes (third row) for TCGA WES (*A*) or PCAWG WGS data (*B*).** Comparison of recall between MuSE 2 and MuTect2 within different clonality for PCAWG WGS data is shown in the last row of (*B*). The calls of each method, and the consensus calls, which are used as a truth set, are pooled from the WES data of 5 patient samples from TCGA (*A*), or the WGS data of 5 patient samples from PCAWG (*B*). The number of consensus calls for a SNV category is included in the x-axis labels.
