# Supplementary material for: Accelerated somatic mutation calling for whole-genome and whole-exome sequencing data from heterogenous tumor samples
Source: Genome Res. 2024 Apr;34(4):633–41. doi: 10.1101/gr.278456.123 (PMC11146589; doi:10.1101/gr.278456.123)
Supplement: Supplement 5 [file Supplemental_Fig_S5.docx]

**
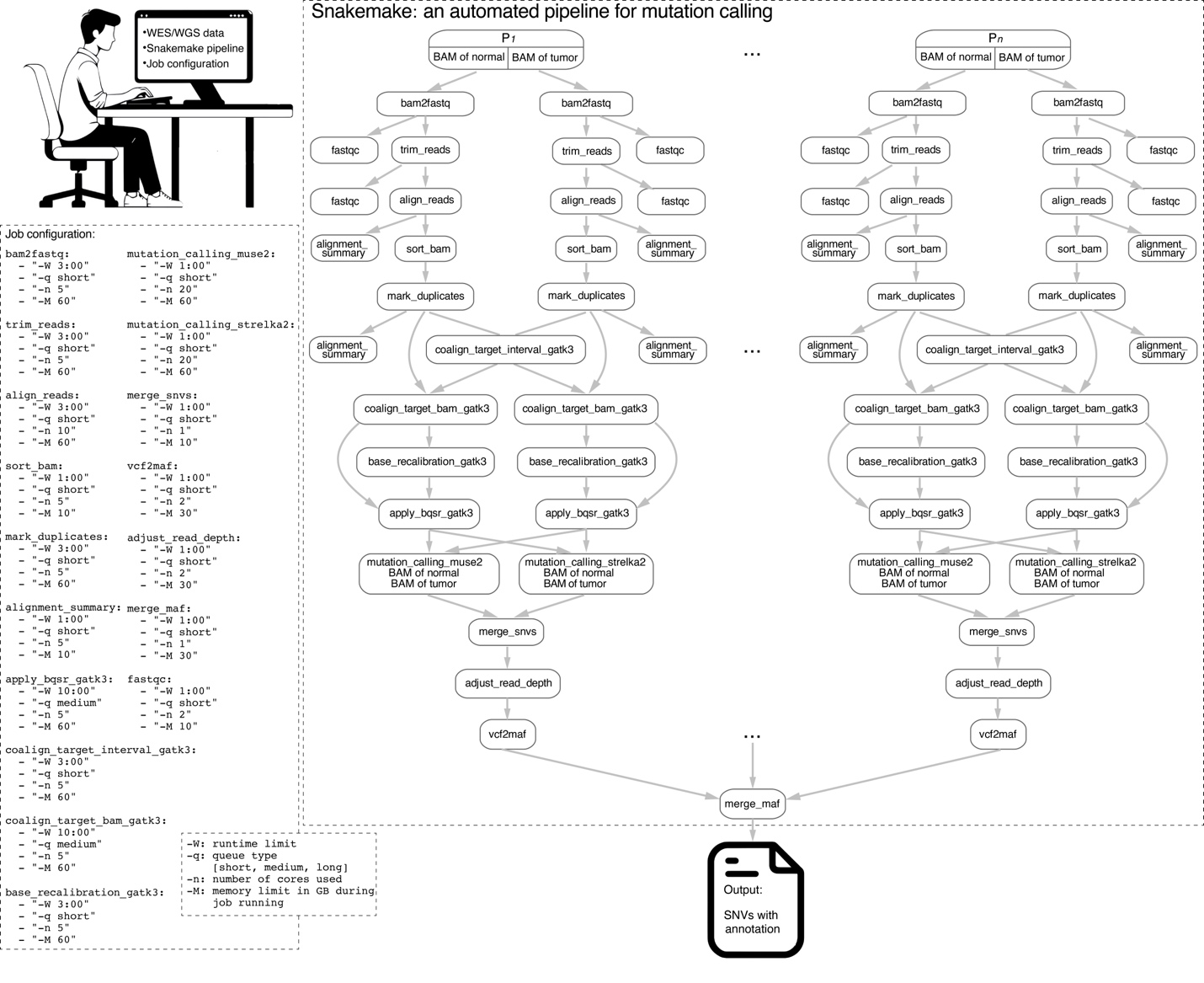
Supplemental Fig. S5 | Illustration of running the Snakemake automated pipeline to call somatic SNVs for WES/WGS data from a patient cohort on a High Performance Computing cluster.**
