# Supplementary material for: Accelerated somatic mutation calling for whole-genome and whole-exome sequencing data from heterogenous tumor samples
Source: Genome Res. 2024 Apr;34(4):633–41. doi: 10.1101/gr.278456.123 (PMC11146589; doi:10.1101/gr.278456.123)
Supplement: Supplement 6 [file Supplemental_Fig_S6.docx]

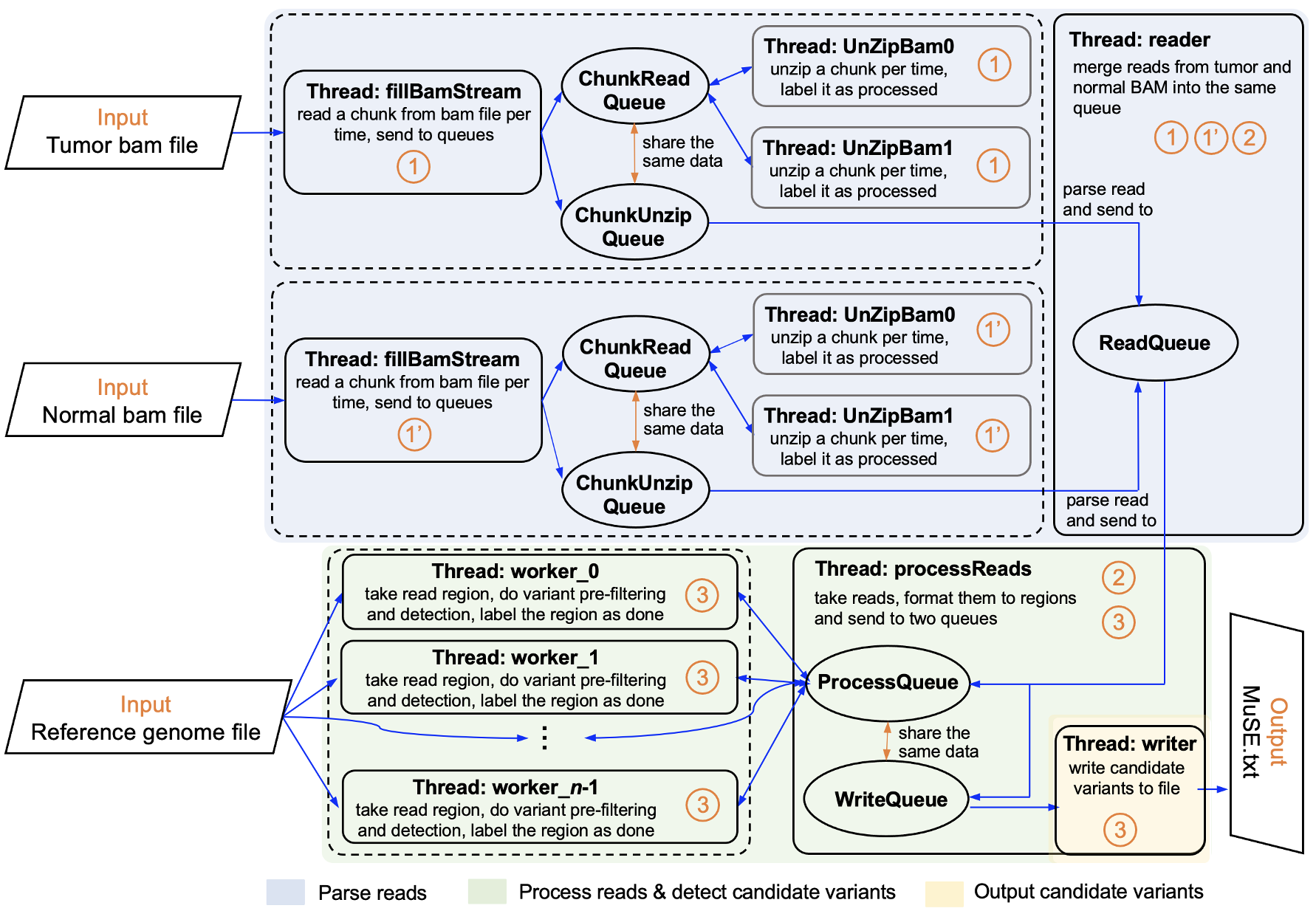


**Supplemental Fig. S6 | Diagram of the parallelization implementation of the ‘call‘ step in MuSE 2.** Diamonds indicate input and output; rectangles indicate threads created for a task described in the box; blue arrows denote data flow. Ellipses are queues exchanging data between the connected threads; orange double-headed arrows denote that the two connected queues sharing the same data; the threads and queues are divided into three groups by their functions as indicated by the shades; circled numbers represent the communication variables between an upstream thread and a downstream thread(s) by which the latter knows if the former completes its jobs; *n* is the number of threads specified for running MuSE 2.
