# Supplementary material for: Accelerated somatic mutation calling for whole-genome and whole-exome sequencing data from heterogenous tumor samples
Source: Genome Res. 2024 Apr;34(4):633–41. doi: 10.1101/gr.278456.123 (PMC11146589; doi:10.1101/gr.278456.123)
Supplement: Supplement 7 [file Supplemental_Table_S1.docx]

**Supplemental Table S1 | Sample information for the benchmarking study.**

|  | Study ID | Sample ID | TCGA filename | Tissue type | Average read depth |
| --- | --- | --- | --- | --- | --- |
| WES | 1 | TCGA-AA-3811 | TCGA-AA-3811-01A-01W-0995-10_  hg19_Illumina_gdc_realn.bam | Tumor | 241X |
|  |  |  | TCGA-AA-3811-10A-01W-0995-10_  hg19_Illumina_gdc_realn.bam | Blood normal | 137X |
|  | 2 | TCGA-AA-A01R | a766b78f-5ed9-447b-ae83-6e06d6f7  a8e1_wxs_gdc_realn.bam | Tumor | 127X |
|  |  |  | 54523ac8-0dd8-4256-b33e-f0a802fe  e3e8_wxs_gdc_realn.bam | Blood normal | 155X |
|  | 3 | TCGA-05-4424 | fc500ff5-24c8-4965-94da-b4afafafe2  dd_wxs_gdc_realn.bam | Tumor | 111X |
|  |  |  | e785fabf-7b0f-49cd-a423-0c6372147  f9b_wxs_gdc_realn.bam | Blood normal | 115X |
|  | 4 | TCGA-EE-A2GD | ecc80084-895a-4810-b1ec-a1039aa  7260d_wxs_gdc_realn.bam | Tumor | 73X |
|  |  |  | b42231d7-56a6-4b1b-8477-406f3b3  2c8e7_wxs_gdc_realn.bam | Blood normal | 101X |
|  | 5 | TCGA-VQ-AA6G | 2ed43bef-b90e-4029-be14-cfdfa58b  ec9a_wxs_gdc_realn.bam | Tumor | 68X |
|  |  |  | 6f381501-d383-443d-9102-96fcce2  ae971_wxs_gdc_realn.bam | Blood normal | 60X |
|  | 6 | TCGA-MH-A55Z | TCGA-MH-A55Z-01A-11D-A26P-  10_Illumina_gdc_realn.bam | Tumor | 66X |
| WGS |  |  | TCGA-MH-A55Z-10A-01D-A26P-  10_Illumina_gdc_realn.bam | Blood normal | 41X |
|  | 7 | TCGA-MO-A47R | PCAWG.cff273a0-2c77-43c2-b0  e8-9ec7d3878d83.bam | Tumor | 45X |
|  |  |  | PCAWG.f4a4dfa6-d5bc-4115-a1  6e-3edd999ad8f9.bam | Blood normal | 34X |
|  | 8 | TCGA-NH-A50T | TCGA-NH-A50T-01A-11D-A28G-  10_Illumina_gdc_realn.bam | Tumor | 75X |
|  |  |  | TCGA-NH-A50T-10A-01D-A28G-  10_Illumina_gdc_realn.bam | Blood normal | 43X |
|  | 9 | TCGA-NH-A50V | TCGA-NH-A50V-01A-11D-A28G-  10_Illumina_gdc_realn.bam | Tumor | 75X |
|  |  |  | TCGA-NH-A50V-10A-01D-A28G-  10_Illumina_gdc_realn.bam | Blood normal | 42X |
|  | 10 | TCGA-PD-A5DF | TCGA-PD-A5DF-01A-11D-A27I-  10_Illumina_gdc_realn.bam | Tumor | 81X |
|  |  |  | PCAWG.af9e5e0d-db6b-4dee-8  4a6-c99462e9f71e.bam | Blood normal | 45X |
|  | 11 | COLO829 | phased_possorted_bamCOLO82  9T.bam (10X platform) | Tumor | 31X |
|  |  |  | phased_possorted_bamCOLO82  9R.bam (10X platform) | Normal | 29X |
|  |  |  | COLO829T_dedup.realigned.bam  (Illumina platform, purity = 100%) | Tumor | 95X |
|  |  |  | illumina_purity75.bam (Illumina  platform, purity = 75%) | Tumor | 92X |
|  |  |  | illumina_purity50.bam (Illumina  platform, purity = 50%) | Tumor | 90X |
|  |  |  | illumina_purity25.bam (Illumina  platform, purity = 25%) | Tumor | 85X |
|  |  |  | illumina_purity20.bam (Illumina  platform, purity = 20%) | Tumor | 83X |
|  |  |  | COLO829R_dedup.realigned.bam  (Illumina platform, purity = 0%) | Normal | 35X |
