# Supplementary material for: Accelerated somatic mutation calling for whole-genome and whole-exome sequencing data from heterogenous tumor samples
Source: Genome Res. 2024 Apr;34(4):633–41. doi: 10.1101/gr.278456.123 (PMC11146589; doi:10.1101/gr.278456.123)
Supplement: Supplement 8 [file Supplemental_Table_S2.docx]

**Supplemental Table S2** **| Precision, recall and F1 scores of mutation calls from Strelka2, MuSE 2 and their intersections for both WES and WGS data.**

| Sample ID | Data type | No. of consensus calls | Intersection of MuSE 2 and Strelka2 | | | Strelka2 | | | MuSE 2 | | |
| --- | --- | --- | --- | --- | --- | --- | --- | --- | --- | --- | --- |
|  |  |  | Precision | Recall | F1 score | Precision | Recall | F1 score | Precision | Recall | F1 score |
| 1 | WES | 2,553 | 0.92 | 0.74 | 0.82 | 0.53 | 0.90 | 0.67 | 0.90 | 0.76 | 0.82 |
| 2 |  | 2,544 | 0.94 | 0.78 | 0.85 | 0.79 | 0.84 | 0.81 | 0.93 | 0.79 | 0.85 |
| 3 |  | 932 | 0.95 | 0.88 | 0.91 | 0.86 | 0.92 | 0.89 | 0.90 | 0.93 | 0.91 |
| 4 |  | 772 | 0.96 | 0.86 | 0.91 | 0.76 | 0.91 | 0.83 | 0.94 | 0.90 | 0.92 |
| 5 |  | 168 | 0.94 | 0.89 | 0.91 | 0.70 | 0.92 | 0.80 | 0.79 | 0.94 | 0.86 |
| 6 | WGS | 7,269 | 0.95 | 0.97 | 0.96 | 0.84 | 0.97 | 0.90 | 0.67 | 0.99 | 0.80 |
| 7 |  | 3,813 | 0.89 | 0.96 | 0.92 | 0.62 | 0.96 | 0.76 | 0.46 | 0.98 | 0.63 |
| 8 |  | 12,494 | 0.90 | 0.96 | 0.93 | 0.77 | 0.96 | 0.86 | 0.72 | 0.99 | 0.83 |
| 9 |  | 19,081 | 0.89 | 0.96 | 0.92 | 0.80 | 0.96 | 0.87 | 0.76 | 0.99 | 0.86 |
| 10 |  | 8,073 | 0.93 | 0.96 | 0.94 | 0.87 | 0.97 | 0.91 | 0.73 | 0.99 | 0.84 |
| COLO829 Illumina |  | 35,543 | 0.89 | 0.98 | 0.93 | 0.76 | 0.98 | 0.86 | 0.79 | 0.99 | 0.88 |
| COLO829 10X |  | 35,543 | 0.87 | 0.94 | 0.91 | 0.71 | 0.97 | 0.82 | 0.70 | 0.96 | 0.81 |
