# Supplementary material for: Accelerated somatic mutation calling for whole-genome and whole-exome sequencing data from heterogenous tumor samples
Source: Genome Res. 2024 Apr;34(4):633–41. doi: 10.1101/gr.278456.123 (PMC11146589; doi:10.1101/gr.278456.123)
Supplement: Supplement 9 [file Supplemental_Table_S3.docx]

**Supplemental Table S3** **| Accuracy of intersect calls from MuSE 2 and Strelka2 or other consensus strategy with additional callers against the benchmark in TCGA WES data.** Note the results shown used individual caller results that did not go through the post-filtering pipeline implemented on the consensus calls by the project of Multi-Center Mutation Calling in Multiple Cancers (MC3) organized by TCGA.

| Sample  ID | Intersection between  MuSE 2 and Strelka2 | | | Consensus from  MuSE 2, Strelka2 and MuTect2 | | | Consensus from  MuSE 2, Strelka2 and VarScan2 | | | Consensus from  MuSE 2, Strelka2 and SomaticSniper | | |
| --- | --- | --- | --- | --- | --- | --- | --- | --- | --- | --- | --- | --- |
|  | Precision | Recall | F1 | Precision | Recall | F1 | Precision | Recall | F1 | Precision | Recall | F1 |
| 1 | 0.92 | 0.74 | 0.82 | 0.60 | 0.91 | 0.72 | 0.86 | 0.81 | 0.83 | 0.92 | 0.74 | 0.82 |
| 2 | 0.94 | 0.78 | 0.85 | 0.86 | 0.83 | 0.85 | 0.90 | 0.82 | 0.86 | 0.88 | 0.83 | 0.86 |
| 3 | 0.95 | 0.88 | 0.91 | 0.90 | 0.95 | 0.92 | 0.90 | 0.92 | 0.91 | 0.93 | 0.90 | 0.91 |
| 4 | 0.96 | 0.86 | 0.91 | 0.83 | 0.94 | 0.88 | 0.90 | 0.93 | 0.92 | 0.91 | 0.89 | 0.90 |
| 5 | 0.94 | 0.89 | 0.91 | 0.88 | 0.96 | 0.92 | 0.80 | 0.94 | 0.86 | 0.79 | 0.91 | 0.85 |
