# Supplementary material for: Accelerated somatic mutation calling for whole-genome and whole-exome sequencing data from heterogenous tumor samples
Source: Genome Res. 2024 Apr;34(4):633–41. doi: 10.1101/gr.278456.123 (PMC11146589; doi:10.1101/gr.278456.123)
Supplement: Supplement 10 [file Supplemental_Table_S4.docx]

**Supplemental Table S4 | Running commands of the benchmarked tools.**

| Tool | Command |
| --- | --- |
| MuSE 2 | MuSE call -O muse -f -n 10 $reference tumor.bam normal.bam  MuSE sump -I muse.MuSE.txt -O muse.vcf -n 20 -E -D dbsnp |
| MuSE 1 | MuSE call -O muse -f $reference tumor.bam normal.bam  MuSE sump -I muse.MuSE.txt -O muse.vcf -E -D dbsnp |
| MuTect2 | gatk Mutect2 -R reference_genome -I tumor.bam -I normal.bam -tumor tumor_id -normal normal_id -O mutect2_raw.vcf.gz  gatk FilterMutectCalls -V mutect2_raw.vcf.gz -R reference_genome -O mutect2_filtered.vcf.gz |
| SomaticSniper | bam-somaticsniper -q 1 -L -G -Q 15 -s 0.01 -T 0.85 -N 2 -r 0.001 -n NORMAL -t TUMOR -F vcf -f reference_genome tumor.bam normal.bam somaticsniper.vcf |
| VarScan2 | samtools mpileup -f $reference -q 1 -B normal.bam tumor.bam > mpileup.pileup  java -jar VarScan.v2.4.1.jar somatic mpileup.pileup varscan_somatic.vcf --mpileup 1 --min-coverage 8 --min-coverage-normal 8 --min-coverage-tumor 6 --min-var-freq 0.10 --min-freq-for-hom 0.75 --normal-purity 1.0 Competing interest statement --tumor-purity 1.00 --p-value 0.99 --somatic-p-value 0.05 --strand-filter 0 --output-vcf  java -jar VarScan.v2.4.1.jar processSomatic varscan.vcf.snp --min-tumor-freq 0.10 --max-normal-freq 0.05 --p-value 0.07 |
| Strelka2 | STRELKA_INSTALL_PATH=  ${STRELKA_INSTALL_PATH}/bin/configureStrelkaSomaticWorkflow.py --normalBam normal.bam --tumorBam tumor.bam --referenceFasta reference_genome --runDir ./  ./runWorkflow.py -m local -j 20 |
