# Supplementary material for: Accelerated somatic mutation calling for whole-genome and whole-exome sequencing data from heterogenous tumor samples
Source: Genome Res. 2024 Apr;34(4):633–41. doi: 10.1101/gr.278456.123 (PMC11146589; doi:10.1101/gr.278456.123)
Supplement: Supplement 11 [file Supplemental_Table_S5.docx]

**Supplemental Table S5** **| Version information of the benchmarked tools.**

| Tool | Version |
| --- | --- |
| MuSE 2 | 2.1 |
| MuSE 1 | 1.0rc |
| MuTect2 | 4.1.9.0 |
| SomaticSniper | 1.0.5.0 |
| Varscan2 | 2.4.2 |
| Strelka2 | 2.9.x |
